# Isotopic evidence for residential mobility of farming communities during the transition to agriculture in Britain

**DOI:** 10.1098/rsos.150522

**Published:** 2016-01-20

**Authors:** Samantha Neil, Jane Evans, Janet Montgomery, Chris Scarre

**Affiliations:** 1 Department of Archaeology, Durham University, South Road, Durham DH1 3LE, UK; 2 NERC Isotope Geosciences Laboratory, Keyworth, Nottingham NG12 5GG, UK

**Keywords:** development of agriculture, Neolithic, sedentism, mobility, strontium, isotope analysis

## Abstract

Development of agriculture is often assumed to be accompanied by a decline in residential mobility, and sedentism is frequently proposed to provide the basis for economic intensification, population growth and increasing social complexity. In Britain, however, the nature of the agricultural transition (*ca* 4000 BC) and its effect on residence patterns has been intensely debated. Some authors attribute the transition to the arrival of populations who practised a system of sedentary intensive mixed farming similar to that of the very earliest agricultural regimes in central Europe, *ca* 5500 BC, with cultivation of crops in fixed plots and livestock keeping close to permanently occupied farmsteads. Others argue that local hunter–gatherers within Britain adopted selected elements of a farming economy and retained a mobile way of life. We use strontium and oxygen isotope analysis of tooth enamel from an Early Neolithic burial population in Gloucestershire, England, to evaluate the residence patterns of early farmers. Our results are consistent with the hypothesis that early farming communities in Britain were residentially mobile and were not fully sedentary. Results highlight the diverse nature of settlement strategies associated with early farming in Europe and are of wider significance to understanding the effect of the transition to agriculture on residence patterns.

## 1. Introduction

The transition from hunting and gathering to farming is often considered to be accompanied by a decline in residential mobility as sedentism is assumed to facilitate economic intensification, leading to population expansion and the development of complex societies (e.g. [1–3]. The agricultural transition in Britain (*ca* 4000–3500 BC) is marked by the importation of non-native species of domesticated animals from continental Europe, evidence for cereal cultivation and the appearance of new traditions of pottery manufacturing, lithic technologies and monument construction. However, both the processes that facilitated the transition and the nature of the first farming systems associated with it remain intensely debated (e.g. [4–8]). Some authors attribute development of farming in Britain to the arrival of settled agriculturalists from continental Europe who practised a similar system of intensive mixed agriculture to that of the Linearbandkeramik (LBK), the first farming systems which developed in central Europe from approximately 5500 BC [9–11]. Arable production is proposed to have been closely integrated with livestock keeping: cultivation is suggested to have taken place in fixed plots with animals being kept close to permanently occupied farmsteads [10,12,13]. Archaeobotanical evidence is considered to rule out shifting cultivation, and agricultural regimes in Early Neolithic Britain are proposed to have been similar to those of the LBK [14]. Cereals are argued to have been a dietary staple [15–17] and, due to the demands of cultivation, it is suggested that the first agriculturalists in Britain were fully sedentary [18,19].

In contrast, other authors argue that unlike the very first agriculturalists in central Europe, early farmers in Britain were not fully sedentary and farming regimes were based on agro-pastoralism [20–22]. These authors suggest that the number of substantial timber buildings so far discovered that date to this period is limited, and question the interpretation that they functioned as permanently occupied farmsteads [23]. Rather than a fully arable economy, subsistence practice is instead proposed to have been predicated on intensive dairying [24–29] and routine exploitation of wild plant species as well as cereals [30–33]. This is in turn considered by some authors to demonstrate that the transition to agriculture occurred through adoption of selected elements of a farming economy by local Mesolithic populations, who retained a mobile way of life thought to be characteristic of hunter–gatherers [8,34,35]. Recent analysis of temporal changes in the robusticity of lower limb bones is also considered to support continued residential mobility during the Neolithic and a gradual, rather than abrupt, transition to sedentism [36]. In Britain, Early Neolithic occupation evidence frequently comprises pits, stakeholes, lithic scatters and middens which are interpreted as the remains of temporary camps that were occupied episodically (e.g. [37–39]). Rather than sedentism, it is therefore suggested that residence patterns were based on ‘tethered mobility’ [40,41], a system of cyclical transhumance in which communities repeatedly moved between favoured occupation sites.

In view of these debates, we applied strontium and oxygen isotope analysis of tooth enamel to evaluate the land use and residence patterns of the first farmers in Britain. Strontium isotope analysis of tooth enamel is a robust and highly reliable technique that is routinely used for geographical provenancing (e.g. [42,43]). Strontium (^87^Sr/^86^Sr) isotope ratios vary with the age and composition of bedrock [44]. Strontium weathers from rocks into soils where it becomes available to plants and enters the human food chain [45]. Tooth enamel is highly resistant to diagenesis (e.g. [46,47]) and as mass-dependent fractionation does not affect conventionally measured ^87^Sr/^86^Sr values [48], strontium isotope ratios in enamel directly reflect the location from which an individual obtained food during the period in which a tooth was mineralizing [49,50]. Comparison of ^87^Sr/^86^Sr values in teeth that form at successive stages of childhood to mapped values in modern vegetation and water (e.g. [51,52]) can therefore be used to evaluate whether an individual was residentially mobile.

The oxygen isotope composition of water also varies geographically with factors such as temperature, latitude and altitude (e.g. [53,54]). In Britain, δ^18^O values of contemporary groundwaters are primarily influenced by rainfall; western Britain receives higher rainfall, and therefore groundwaters have higher δ^18^O values than those in eastern Britain [55]. A statistically significant difference in the mean δ^18^O_phosphate_ values measured in tooth enamel of multi-period archaeological populations buried in western Britain (18.2‰±1‰, 2*σ*) from those in eastern Britain (17.2±1.3‰, 2*σ*) is considered to reflect the underlying geographical variation in the oxygen isotope composition of local drinking water between the two areas [56]. It is argued that occupation of these different regions of Britain is associated with 95% ranges of 17.2 to 19.2‰ and 15.9 to 18.5‰, respectively (ibid.). These ranges were determined using isotope analysis of the phosphate (PO_4_^3−^) fraction of tooth enamel. However, the carbonate (CO_3_^2−^) fraction is equally suitable for analysis and, as the δ^18^O values in the δ^18^O_phosphate_ and δ^18^O_*carbonate*_ fractions are considered to be well correlated, conversion between the two can be undertaken using the equation developed by Chenery *et al.* [57] (see Material and methods). Interpretation of δ^18^O results must, however, give consideration to the potential influence of culturally mediated behaviour, such as culinary practice (e.g. stewing foods and brewing) [58] or consumption of fluids that have undergone fractionation through biological processes (e.g. breast milk or cow’s milk) [59–65] on the oxygen isotope composition of ingested fluids.

Isotope analysis of the structural carbonate fraction of enamel simultaneously yields carbon isotope ratios (δ^13^C_*carbonate*_) which provide additional dietary information. The use of carbon isotope analysis for this purpose exploits the large variation in natural abundance of δ^13^C between plants that use the two dominant (C_3_ or C_4_) photosynthetic pathways during fixation of CO_2_ energy and variation in δ^13^C values between terrestrial C_3_ and marine ecosystems (e.g. [66,67]). Current understanding of dietary composition in the European Neolithic is based on analysis of δ^13^C and δ^15^N values in bone collagen, which predominantly reflect the protein component of the diet and support the exploitation of C_3_ terrestrial sources of protein during the Early Neolithic in Britain (e.g. [68–70]). In contrast, δ^13^*C*_*carbonate*_ values in bioapatite reflect the isotope composition of the diet as a whole, including lipids and carbohydrates [71,72]. Individuals who obtain all of their diet from C_3_ terrestrial sources may be predicted to have δ^13^C_*carbonate*_ values between approximately −17.0 to −14.0‰ [73,74].

**Figure 1.**
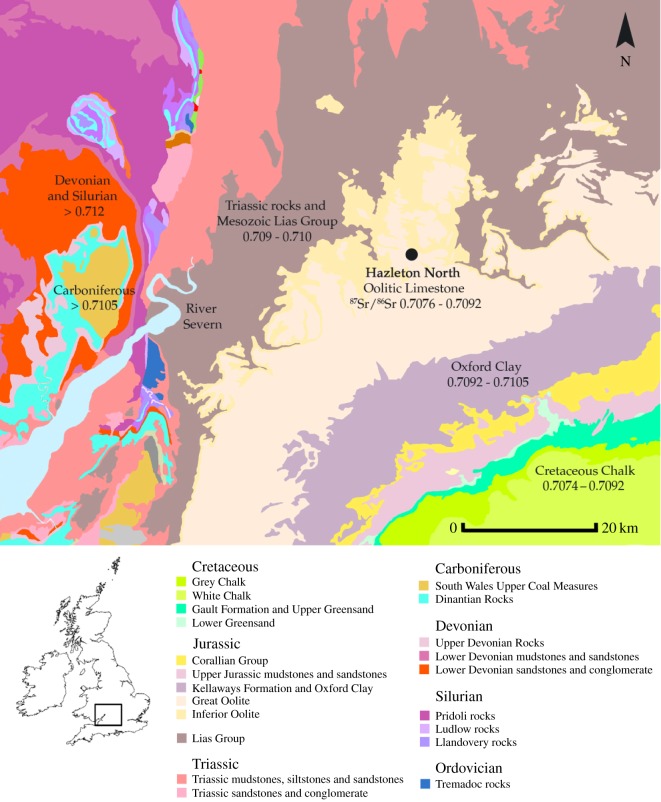
Map of bedrock geology illustrating sites and locations discussed in the text. Based on British Geological Survey and Ordnance Survey map data, reproduced with permission of the British Geological Survey and Ordnance Survey, © NERC/Crown copyright 2015. Bioavailable ^87^Sr/^86^Sr ranges associated with different lithologies are based on measured values by Evans *et al.* [51], Warham [52] and Chenery *et al.*[78].

Thirty-eight teeth, including the consecutively mineralizing molars of 18 different individuals, were analysed to obtain strontium, oxygen and carbon isotope ratios (see Material and methods and table 1). The sampled population derives from Hazleton North long cairn, one of the few Early Neolithic monuments in Britain which has been completely excavated to modern standards [75,76]. The monument, which is estimated to have been constructed between 3710 and 3655 cal. BC and used for burial over at least two to three generations (95% probability, OxCal v. 3.5) [77], is situated in the Cotswold region of England (figure 1). In addition to analyses undertaken on individuals buried in chambers on the north and south sides of the monument, a tooth from a small scatter of human remains found stratified underneath the long cairn that is considered to represent the earliest dated burial activity at the site (ibid.) was also analysed. The presence of a hearth, post-holes, a midden and evidence for cultivation directly beneath the monument are argued to indicate that it was constructed at a site previously used for occupation [76,79]. Samples of enamel from the three main domesticated species found in the midden (cattle, sheep/goat and pig) [80] were also taken for analysis.

**Table 1.** Strontium isotope ratios, strontium concentrations and δ^18^O_carbonate_ and δ^13^C_carbonate_ values in enamel of humans and animals from Hazleton North. Approximate age at death is based on tooth eruption after Rogers [75], pp. 190–191; L = left, R = right; mandibular first, second and third permanent molar teeth are designated as M1, M2 and M3, respectively; second permanent premolar teeth are designated as PM2; first mandibular central permanent incisor teeth designated as LI1.

sample number	location	context/ box number	age at death	tooth	^87^Sr/^86^Sr	Sr ppm (mg kg^−1^)	δ^13^C_carbonate_ ‰ VPDB	δ^18^O_carbonate_ ‰ VPDB	δ^18^O_carbonate_ ‰ VSMOW	δ^18^O_phosphate_ ‰ VSMOW
10 414/individual G	north chamber basal fill	336	3–4 years	mandibular LM1	0.71027	54	−16.0	−3.8	27.0	18.2
10494	south chamber fill	412	adult	maxillary LM3	0.70963	49	−15.1	−4.0	26.8	18.0
11456	south chamber fill	412	adult	mandibular LM2	0.71036	67	−15.2	−4.2	26.6	17.8
				mandibular LM3	0.71016	58	−16.7	−4.4	26.4	17.6
11 903	pre-cairn; SW quad cell S	211	unknown	loose premolar	0.70866	45	−16.0	−3.5	27.3	18.5
12527	south chamber	453	6–9 years	mandibular RM1	0.70833	62	−16.1	−2.5	28.3	19.5
3596	south entrance fill	354	adult	mandibular RPM2	0.70853	47	−16.1	−3.8	27.0	18.2
3793	south entrance fill	354	adult	mandibular RM1	0.70818	63	−14.9	−3.1	27.8	19.0
				mandibular RM2	0.70818	44	−14.7	−3.8	27.0	18.2
				mandibular RM3	0.71033	101	−15.7	−3.1	27.7	18.9
3831	south entrance fill	354	adult	mandibular RM2	0.71036	144	−15.0	−4.0	26.8	18.0
				mandibular RM3	0.70838	35	−16.3	−3.8	27.0	18.2
4077/4169	south entrance fill	354	adult	mandibular RM1	0.70835	37	−16.0	−2.9	28.0	19.2
				mandibular RM2	0.70827	30	−15.5	−3.7	27.1	18.3
				mandibular RM3	0.70806	24	−16.3	−4.2	26.6	17.8
4118	north entrance fill	267	adult	mandibular LI1	0.71027	65	−15.4	−3.5	27.3	18.5
4786	south chamber passage	187	adult	maxillary LM2	0.70839	48	−14.7	−3.1	27.7	18.9
4806/7387	south chamber passage	323	adult	mandibular LM1	0.70887	39	−16.4	−3.0	27.8	19.1
5037/Skeleton 1	north entrance	267	adult	maxillary LM1	0.70797	22	−16.5	−3.9	26.9	18.1
				maxillary RM2	0.70804	22	−16.0	−3.7	27.1	18.3
				maxillary RM3	0.70825	27	−15.9	−3.8	27.0	18.1
5880	north chamber basal fill	336	adult	mandibular LM1	0.70957	85	−15.2	−3.2	27.6	18.8
				mandibular LM2	0.70912	52	−15.8	−3.2	27.6	18.8
				mandibular LM3	0.70888	40	−16.1	−3.8	27.0	18.2
7386/6815	south chamber passage	323	adult	mandibular LPM2	0.70838	45	−15.5	−4.4	26.4	17.6
7656	south chamber passage	323	adult	mandibular RM1	0.70794	41	−15.2	−3.0	27.2	19.1
				mandibular RM2	0.70855	55	−14.5	−3.6	27.2	18.4
				mandibular RM3	0.70813	32	−16.4	−3.6	27.2	18.4
8701/individual E	south chamber fill	412	12–15 years	mandibular RM1	0.70807	40	−16.1	−3.9	26.9	18.1
8751	south chamber fill	412	adult	mandibular LM2	0.70804	37	−15.8	−3.7	27.1	18.2
				mandibular LM3	0.71066	84	−16.0	−3.3	27.5	18.7
8974	south entrance fill	353	adult	mandibular LM2	0.70962	76	−15.9	−4.4	26.4	17.6
				mandibular LM3	0.71120	88	−15.6	−4.0	26.8	18.0
9025	north chamber fill	435	adult	mandibular LM1	0.71262	74	−15.6	−3.2	27.6	18.8
9951	south chamber fill	412	9–10 years	mandibular LM1	0.70810	62	−15.5	−4.0	26.8	18.0
HBG HN82/15 374 cow	pre-cairn/NW quad cell R	211/box 23	unknown	loose molar tooth	0.71059	180				
HBG HN82/16 065 pig	pre-cairn/NW quad cell R	211/box 29	unknown	maxillary LM3	0.70774	82				
HBG HN82/18 304 sheep/goat	pre-cairn/SW quad cell S	211/box 31	unknown	loose molar tooth	0.70821	216				

The lithology at the site and in the surrounding Cotswold region of Gloucestershire is composed of Oolitic limestone [81,82] a marine carbonate rock which has an ^87^Sr/^86^Sr value equivalent to seawater in the Middle Jurassic period (0.7068–0.7073) [83]. However, the bioavailable ^87^Sr/^86^Sr range on Oolitic limestone is also influenced by rainwater [84]. In an island facing the Atlantic such as Britain, the value of rainwater is close to that of seawater, which throughout the Holocene has had a ratio close to 0.7092 [83,85]. Due to the combination of these two sources of strontium, samples from plants and waters on Oolitic limestones in the Cotswolds give ^87^Sr/^86^Sr values between 0.7076 and 0.7092, with a mean of 0.7086±0.0004 (1*σ*, *n*=17) [52,78,86]. A sedentary self-sufficient population subsisting solely on resources obtained from a homogeneous lithological unit such as Oolitic limestone would be predicted to plot on a diagonal mixing array between two sources of dietary strontium (end-members): the ratio bioavailable on that lithological unit and that of rainwater [42,87]. Had the sampled population derived all their resources locally, cultivating fields and keeping herds of animals around a permanently occupied settlement at the site or in the surrounding Cotswold region of England, they would be expected to plot between the minimum value bioavailable on Oolitic limestone (0.7076) and rainwater (∼0.7092).

## 2. Material and methods

### 2.1 Sample selection

The human burial assemblage from Hazleton North consists of disarticulated and co-mingled human remains. Care was therefore taken to avoid the potential for duplication of isotope results through inadvertent sampling of antimeres which could belong to the same individual. Teeth that remained *in situ* in left-sided mandibular fragments were therefore selected for sampling. Teeth in right-sided mandibular fragments were not sampled unless the refitting left-hand side of the dentition was present. Maxillary teeth were only used if the re-fitting mandible belonging to the individual was present. In total, 18 different individuals (14 adults and four pre-adults) were sampled. In addition to the 18 discrete individuals sampled by this project, two chips of core enamel taken during sampling of maxillary dentition by a project unrelated to this study were also analysed to obtain isotope ratios: 4786 (LM2) and 10 494 (LM3). Like the human assemblage, the pre-cairn animal assemblage from Hazleton North is also highly fragmentary. Cranial remains are dominated by loose teeth which cannot be assigned to specific individuals [80]. One tooth from each of the three main domesticated species (cattle, sheep/goat and pig) present in the pre-cairn assemblage (ibid.) was sampled in order to compare ^87^Sr/^86^Sr values with those of the human group.

Due to the fragmentary nature of the assemblage and disarticulation of cranial remains from other skeletal elements, the sex of the majority of sampled individuals cannot be stated with confidence. Only one individual, Skeleton 1, was found in a virtually complete fully articulated state and may be sexed as male [75]. Where available, information on the approximate age of the individuals, as determined by dental eruption, is provided in table 1, after Rogers [75], pp. 190–191. Wherever present, consecutively mineralizing molar teeth were selected in order to examine the variability in isotope ratios between teeth that form at different stages of childhood. Development of the crown of the first permanent adult molar commences *in utero*, just prior to birth, and completes by approximately 4.5±0.5 years of age, while the second molar crown forms between 2.5±0.5 years and 8.5±0.5 years of age [88,89]. The timing of third molar formation is most variable [90], with initial cusp formation taking place at approximately 8.5±0.5 years and crown completion by 14.5±0.5 years [88]. Strontium and oxygen isotope analysis was conducted on samples of bulk enamel, and isotope ratios therefore represent the weighted average of the sources to which the individual was exposed during the period the tooth was mineralizing. As the process of enamel formation is highly complex (e.g. [89]), and as strontium may have an extended residence time within the body prior to its incorporation in enamel [42], it is currently uncertain that greater chronological resolution can be achieved by serial sampling of human tooth enamel.

### 2.2 Sample preparation and laboratory analysis

Teeth were processed following procedures developed by Montgomery [49]. Surface enamel was thoroughly abraded using a tungsten carbide dental burr. Enamel chips were then cut using a flexible diamond-edged rotary saw and surfaces again mechanically cleaned using a tungsten carbide dental burr to remove any adhering dentine. An enamel chip of approximately 20–30 mg in weight from each tooth was taken for strontium isotope analysis and of approximately 10 mg in weight for oxygen isotope analysis. Dental saws and burrs were cleaned ultrasonically for 5 min and rinsed three times in high purity de-ionized water between preparation of samples.

### 2.3 ^87^Sr/^86^Sr analysis

Samples were transferred in clean sealed containers to the Class 100, HEPA-filtered laboratory facilities at the Natural Environment Research Council Isotope Geosciences Laboratory (Keyworth, Nottingham, UK). Enamel chips were cleaned ultrasonically and rinsed in high purity water (Millipore Alpha Q). They were then dried, weighed into pre-cleaned Teflon beakers and spiked with a known amount of ^84^Sr tracer solution to obtain strontium concentrations. Each sample was dissolved in Teflon distilled 8 M HNO_3_. Samples were converted to chloride using 6 M HCl, taken up in titrated 2.5 M HCl and pipetted onto ion-exchange chromatography columns. Strontium was separated with Dowex^®^ (AG50-X8) resin (200–400 mesh). Procedural blanks were below 150 pg. Samples were loaded on to Re filaments using a method adapted from Birck [91]. Strontium isotope composition and concentrations were then determined by thermal ionization mass spectroscopy using a ThermoTriton automated multi-collector mass spectrometer. To correct for fractionation during the process of mass spectrometry, ^87^Sr/^86^Sr values are normalized to the accepted value for ^88^Sr/^86^Sr = 0.1194. During the period of this study, the machine gave a value for the international standard for ^87^Sr/^86^Sr (NBS 987) of 0.710253±0.000012 (2*σ*, *n*=350). An estimate of the reproducibility of strontium concentration (Sr ppm) is provided by replicate analysis of an aliquot of bone standard solution (NIST1486), which gave 7.22±0.27 ppm (±3.75%, 1*σ*, *n*=16).

### 2.4 δ^18^O and δ^13^C analysis

Initial preparation of core enamel chips for δ^18^O and δ^13^C analysis was undertaken using the same methods employed above for strontium isotope analysis. Samples were then transferred as clean core enamel chips to the Natural Environment Research Council Isotope Geosciences Laboratory where they were powdered. Oxygen (δ^18^O_carbonate_) and carbon (δ^13^C_carbonate_) isotope ratios in the carbonate fraction of enamel were determined using approximately 3 mg of clean powdered enamel following the method outlined in Chenery *et al.* [57]. Isotope ratios are reported as delta (δ) values, in parts per thousand (per mil; ‰) normalized to the VPDB scale using an in-house carbonate reference material, Keyworth Carrera marble (KCM), which is calibrated against NBS19 certified reference material. Analytical reproducibility for this run of KCM was ±0.09‰ (1*σ*, *n*=14) for δ^18^O and for δ^13^C±0.04‰ (1*σ*,*n*=14). δ^18^O_carbonate_ values were normalized to the VSMOW scale using the equation of Coplen [92] (VSMOW=1.03091×δ^18^O VPDB + 30.91). Conversion between δ^18^O_carbonate_ to δ^18^O_phosphate_ was then undertaken using the regression equation of Chenery *et al.* [57] (${\text{δ}}^{18}{\text{O}}_{\mathrm{phosphate}}=1.0322\times {\text{δ}}^{18}{\text{O}}_{\mathrm{carbonate}}-9.6849$). The error involved in calculating δ^18^O_phosphate_ is considered to be low (0.28‰, 1*σ*, ibid.).

## 3. Results

**Figure 2.**
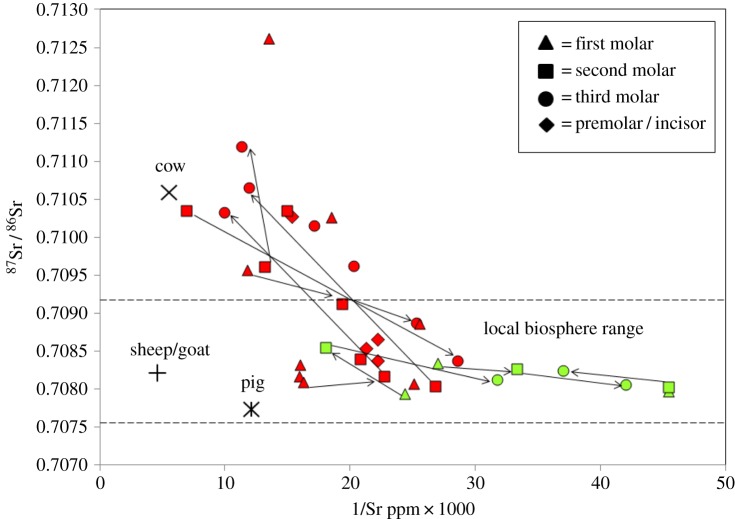
Plot of strontium isotope ratio versus the inverse of concentration (1/Sr ppm × 1000) for individuals and animals. Dashed lines delineate the approximate ^87^Sr/^86^Sr biosphere range available on Oolitic limestone. Light green symbols indicate individuals who can be interpreted as sedentary and red symbols denote the rest of the population. Tooth types are denoted by the key in the upper right of the diagram. Cow, sheep/goat and pig labelled within the diagram are from pre-cairn contexts. 2*σ* errors for ^87^Sr/^86^Sr are within the symbol.

The majority of the population plot on a diagonal array in which ^87^Sr/^86^Sr increases with elemental concentration between a lower value less than 0.7085 and an upper value more than 0.7105. Adjacent molar teeth of individuals in the group also exhibit a shift in strontium isotope ratio and concentration between a lower value less than 0.7085 and an upper value more than 0.7105, or vice versa (figure 2). Isotope ratios do not vary with burial context: individuals from chambers on both the north and south side of the monument plot on the same array. Only one individual appears to be an outlier from the strontium isotope array with a value higher than 0.7125. Three individuals have an ^87^Sr/^86^Sr value that is consistent with the local biosphere ^87^Sr/^86^Sr range on all three of their consecutively mineralizing molar teeth (highlighted in light green, figure 2).

**Figure 3.**
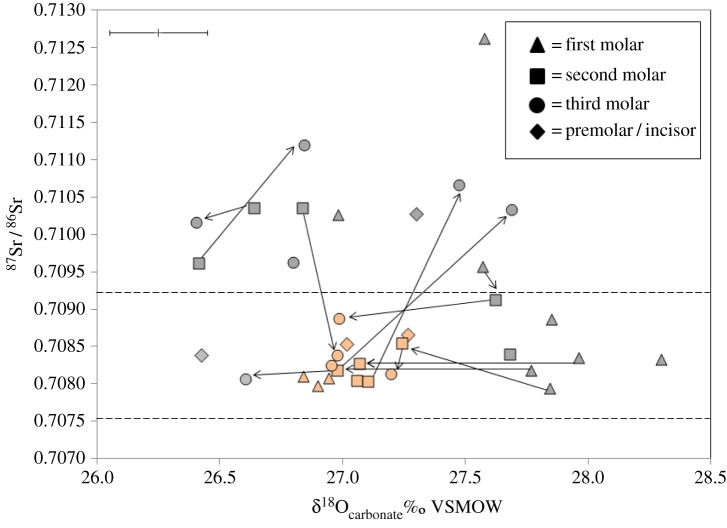
Plot of ^87^Sr/^86^Sr and δ^18^O_carbonate_ results. Dashed lines denote the local ^87^Sr/^86^Sr biosphere range. Teeth highlighted in orange have ^87^Sr/^86^Sr values that are comparable to the local biosphere range and δ^18^O_carbonate_ values that cluster close to 27.0‰. Tooth types are illustrated within the key in the upper right of the diagram. 2*σ* errors for ^87^Sr/^86^Sr are within the symbol. Analytical error for δ^18^O_carbonate_ is shown as ±0.2‰ (2*σ*).

δ^18^O_carbonate_ values range between 26.4 and 28.3‰, with a mean of 27.1±0.4‰ (*n*=35,1*σ*). With the exception of first molar teeth, which more frequently exhibit oxygen isotope ratios higher than 27.5‰ (figure 3), the majority of teeth with ^87^Sr/^86^Sr values near to 0.7085 have δ^18^O_carbonate_ values that plot in a cluster close to 27.0‰. In contrast, teeth with higher strontium isotope ratios (more than 0.7105) that plot above the local biosphere range exhibit a less constrained range of δ^18^O_carbonate_ values. δ^13^C_carbonate_ values of the sampled human population range between −16.6 and −14.5‰ (mean 15.6±0.5‰, *n*=35, 1*σ*; table 1) and therefore fall within the range of values expected for a diet dominated by C_3_ terrestrial sources. Animals sampled from the pre-cairn contexts (figure 3) exhibit a comparable range of ^87^Sr/^86^Sr values to the human group. While the sheep/goat and pig have strontium isotope ratios comparable to the local biosphere range, the cow has a value which is higher than 0.7105. The herbivores that were sampled exhibit higher strontium concentrations than the human population. This is consistent with the progressive discrimination against strontium which results from bio-purification of calcium with increasing trophic level within a food chain (e.g. [93,94]).

## 4. Discussion

A sedentary self-sufficient population subsisting solely on resources obtained from a homogeneous lithological unit such as Oolitic limestone would be predicted to plot on a diagonal mixing array between two sources of dietary strontium (end-members), the ratio bioavailable on that lithological unit and that of rainwater [42,87]. The majority of individuals do plot on a diagonal array, which indicates that they derived dietary strontium from two dominant sources (dietary end-members) that were incorporated in differing proportions during tooth mineralization [ibid.]. However, the strontium isotope array does not conform to that predicted for a sedentary self-sufficient population who had subsisted solely on locally bioavailable resources. One of the two dietary sources exploited by the group had a ^87^Sr/^86^Sr value close to 0.7085 and is therefore comparable to the local ^87^Sr/^86^Sr biosphere range. However, the other dietary end-member (more than 0.7105) is not [51,52,78]. In southern Britain lithologies that routinely give measured biosphere ^87^Sr/^86^Sr values below 0.7085 are geographically separated from those that give values higher than 0.7105, with the exception of the Lizard Peninsula in Cornwall where a small area of serpentinite crops out next to Devonian rocks, approximately 300 km away from Hazleton North [51]. With the exception of the latter area, all current measured ^87^Sr/^86^Sr biosphere values suggest that strontium isotope ratios below 0.7085 and values above 0.7105 are not routinely bioavailable in close proximity in southern Britain. Therefore, to generate the array seen in figure 2, a population who inhabited southern Britain would need to have sourced their diet from at least two different geographical locations. In the absence of any evidence for a market economy during this period to suggest communities derived a significant component of their diet through trade, the strontium isotope array is consistent with movement of individuals between different localities to obtain dietary resources. The closest proximal area to the site where ^87^Sr/^86^Sr values above 0.7105 are routinely bioavailable is more than 40 km to the west or southwest. Plants and waters on lithologies of Carboniferous, Devonian or Silurian age in areas of southwestern Britain such as Gloucestershire, Herefordshire or Worcestershire routinely give values higher than 0.7105, although areas further afield, for example in Wales or Somerset, cannot be excluded [51,78]. The interpretation that the group routinely derived dietary strontium from at least two separate locations is also supported by strontium isotope results from adjacent molar teeth which plot on the same strontium isotope array. Several individuals in the group exhibit a shift in ^87^Sr/^86^Sr values from the upper (more than 0.7105) to the lower end-member (less than 0.7085), or vice versa (illustrated by arrows in figure 2). This shift in values between consecutively mineralizing molar teeth is consistent with regular movement backward and forward between at least two different geographical locations.

δ^18^O_carbonate_ results may also support the interpretation that the group derived their diet from more than one location. The majority of teeth with ^87^Sr/^86^Sr values that are comparable to the local biosphere range have δ^18^O_carbonate_ values that plot in a cluster close to 27.0‰. Individuals who plot within this cluster appear to have derived ingested fluids from a source which conferred a very similar oxygen isotope value. Deviation in values from the cluster, which appears to represent one of the dietary sources exploited by the sampled group, could be a consequence of localized variation in the oxygen isotope composition of groundwaters between the different geographical locations used by the population. Adjacent molar teeth of different individuals within the sampled group exhibit a shift in oxygen isotope values backward and forward, into and out of this cluster, with those teeth that have higher strontium isotope ratios (more than 0.7105) being associated with a less constrained range of δ^18^O_carbonate_ values. First molar teeth, which begin to form just prior to birth [88,89], more frequently plot with δ^18^O_carbonate_ values that are higher than 27.5‰ (figure 3) and it is possible that values within these teeth may be influenced by consumption of breast milk, which has a higher δ^18^O value relative to meteoric water as a result of the metabolic fractionation that occurs in the mother’s body [63–65]. The mean δ^18^O_phosphate_ value of second and third molar teeth 18.2±0.4‰ (*n*=20, 1*σ*) is, however, comparable to that which has been proposed by Evans *et al.* [56] to represent occupation of the western side of Britain (18.2‰±1‰, 2*σ*) and could support the interpretation that the group routinely moved around lithologies within this region.

The majority of the population, both adults and children of different ages at death, and consecutively mineralizing molars of different individuals, have ^87^Sr/^86^Sr values which conform to the same strontium isotope array. As such it is highly likely that the sampled group participated in a very similar residential routine throughout the period to which the burials are dated, over at least two to three generations during the thirty-seventh century BC [77]. The tooth from pre-cairn contexts (table 1) plots within the cluster of individuals who have teeth with ^87^Sr/^86^Sr values comparable to the local biosphere range and δ^18^O_carbonate_ values close to 27.0‰.This individual could therefore have derived their diet from one of the locations that was exploited by the population who were buried within the cairn. The ^87^Sr/^86^Sr value of the lower dietary end-member exploited by the human burial population (less than 0.7085) is consistent with the local bioavailable range and, in conjunction with the presence of a hearth, midden and evidence for cultivation beneath the monument [76,77], supports the hypothesis that the site itself was one of the two locations occupied during childhood and adolescence by the population who were subsequently buried within the cairn. Occupation of other areas in southern Britain, such as the Cotswolds, or Cretaceous Chalk, which afford a similar bioavailable range [51], cannot be excluded (figure 1). However, the inference that the site itself may have been of significance within the residential tradition of the group who were later buried in the cairn is further supported by the presence of fragments of worked quartzitic sandstone found in pre-cairn contexts. These fragments were imported to the site from at least 40 km away. They derive from lithologies of Carboniferous or older age [82] which routinely give bioavailable ^87^Sr/^86^Sr values comparable to those that provided the upper end-member (more than 0.7105) for the group buried in the cairn [51,78]. Strontium isotope ratios of animals in the midden below the cairn also appear to reflect the residential regime of the human population in the cairn. The animals in the midden were raised at more than one geographical location. They have ^87^Sr/^86^Sr values that are comparable to the upper and lower dietary end-members exploited by the human group and thus are consistent with those of the two areas used by the population who were buried at the site.

Only three individuals in the sampled human population (highlighted in light green, figure 2) possess values below 0.7085 on each of their consecutively mineralizing molar teeth. This could be consistent with sedentism. Although it is possible that these individuals moved between different areas which afford the same ^87^Sr/^86^Sr bioavailable range (e.g. between Oolitic limestone and Cretaceous Chalk), the presence of similar values on adjacent molar teeth may support the interpretation that they occupied one of the locations exploited by the group for a longer period during their early life. Unlike these individuals, the majority of the sampled population do not exhibit values that are consistent with permanent occupation of the same location during early life, or with ‘radial mobility’ [11], brief visits to temporary outlying camps from a single permanent settlement. Ratios in bulk enamel represent the weighted average of all sources of strontium to which the individual had been exposed during the period the tooth was mineralizing [42]. To gain a value higher than ^87^Sr/^86^Sr 0.7105, an individual would need to have derived a significant part of their diet from an area of radiogenic geology, more than would be obtained by a brief visit away from an area with a value below 0.7085. In addition, the regular shift in values exhibited by individuals between adjacent molar teeth, from 0.7105 to 0.7085 or vice versa, is also consistent with a change in location between the two areas used by the group. The possibility that the array seen in figure 2 represents a migrant population who had been fully sedentary at a distant location, for example on the continent, where lithologies that provided biosphere values above 0.7085 and below 0.7105 cropped out close together (i.e. within the same field system), should also be considered. However, evidence for cultivation and occupation beneath the cairn [76,79] in conjunction with evidence for the sourcing of artefacts (above) and strontium isotope results from animals in pre-cairn contexts support the hypothesis that the location at which the cairn was constructed was of pre-existing importance within the residential tradition of a group who inhabited southern Britain.

Results therefore support the model of ‘tethered mobility’ proposed by Whittle [40,41], p. 21, 43, a settlement system in which individuals repeatedly moved between favoured occupation sites. Strontium isotope ratios in tooth enamel are a reflection of sources to which people were exposed during early life and as such the results could be compatible with routine movement of individuals during childhood and adolescence between two communities living in different areas. Alternatively, the array seen in figure 2 could be consistent with a system of cyclical transhumance in which members of the community routinely moved between pastures with their livestock, between for example the Oolitic limestone in the vicinity of the site and older lithologies to the west of the river Severn, as the animals sampled possess ^87^Sr/^86^Sr values comparable to those exhibited by the human group and reflect exploitation of at least two different geographical locations.

The results may therefore be contrasted with the system of sedentary intensive mixed farming that has been proposed to characterize the LBK (*ca* 5500–4900 BC), in which arable production was closely integrated with livestock keeping at permanently occupied hamlets and villages [95,96]. While there is evidence for cultural variability in lifeways during the LBK (e.g. [97–100]), the majority of strontium isotope results are considered to support a system of inherited male access to local plots of land that were located close to permanent settlements [101], with livestock being routinely kept near to the homebase [102,103]. Our results from Britain contrast with this. The majority of individuals in the sampled group from Hazleton North did not derive dietary resources from sedentary intensive mixed farming at a single geographical location. Results are instead consistent with individuals having participated in a regular routine of residential mobility between different geographical locations.

## 5. Conclusion

Agricultural development across Europe has been proposed to have been predicated on a similar system of sedentary intensive mixed farming, with close integration of arable production and livestock keeping at permanently occupied settlements [10,11,13,18,104]. However, while there is strong evidence that this may have provided the basis for the earliest farming systems in central Europe during the sixth millennium BC, the argument that this model can be used as a template for subsequent developments in Britain during the fourth millennium has been challenged [8,20] due to the highly varied nature of occupation evidence which suggests that early farming communities in Britain may have been residentially mobile (e.g. [105–107]). Our results are consistent with the hypothesis that individuals within early farming communities in Britain participated in a regular routine of mobility between different geographical areas and were not fully sedentary. While some of those within the sampled group may have permanently occupied a single location, the majority do not have values consistent with sedentism. Individuals routinely moved between different geographical locations. Evidence for residential mobility need not, however, imply continuity from the local Mesolithic within Britain, as the presence of similar settlement systems on the continent during the fourth millennium BC cannot be ruled out. The results do, however, highlight the diverse nature of residence patterns associated with early agriculture in Europe and provide evidence for cultural variability in settlement practices during the development of farming.

## Data accessibility

The datasets supporting this article are included in table 1 within the manuscript.
